# Association between preoperative fear of falling and mortality after hip fracture surgery in older adults: a post-hoc analysis of a prospective cohort

**DOI:** 10.1007/s41999-026-01597-6

**Published:** 2026-09-22

**Authors:** Feng Gao, Yimin Chen, Mingjian Bei, Yixiao Chen, Ruijiang Li, Shanbin Xu, Liunan Chen, Zhelun Tan, Gang Liu, Minghui Yang, Xinbao Wu

**Affiliations:** 1 Department of Orthopaedics and Traumatology, Beijing Jishuitan Hospital, Capital Medical University, Beijing, 100035 China; 2 Department of Orthopaedics and Traumatology, Peking University Fourth School of Clinical Medicine, Beijing, 100035 China; 3 Department of Orthopaedics and Traumatology, National Center for Orthopaedics, Beijing, 100035 China

**Keywords:** Older adults, Hip fractures, Mortality, mFES, Fear, Falling

## Abstract

**Purpose:**

To examine whether preoperative fear of falling, measured with the modified Falls Efficacy Scale (mFES), is associated with short-term (30-day) and long-term (3-year) postoperative mortality in older adults undergoing hip fracture surgery.

**Methods:**

This post-hoc analysis was based on a prospective observational cohort study. Patients aged ≥ 65 years who underwent surgery for hip fractures between November 2018 and November 2019 were included. Fear of falling was assessed at admission using the mFES. All-cause mortality was assessed by telephone follow-up at 30-day, 120-day, 1-year, and 3-year after admission. Logistic regression and Cox proportional hazards models were used to evaluate the associations between mFES score and short-term and long-term mortality.

**Results:**

A total of 1092 patients were enrolled. The median mFES score was 8.04 (IQR 5.75–10.00). In the multivariable logistic regression analysis, higher mFES scores were independently associated with lower odds of 30-day mortality (adjusted OR 0.780, 95% CI 0.625–0.974, P = 0.028). In the multivariable Cox proportional hazards model, higher mFES scores also remained independently associated with lower long-term mortality risk during the 3-year follow-up period (adjusted HR 0.925, 95% CI 0.874–0.979, P = 0.007).

**Conclusion:**

Lower preoperative mFES scores were independently associated with increased postoperative mortality in older adults undergoing hip fracture surgery. The association was strongest for 30-day mortality, while a weaker but persistent association with long-term mortality was also observed over the 3-year follow-up period.

## Key summary points

**Aim:**

To investigate the association between preoperative fear of falling measured with the modified Falls Efficacy Scale (mFES) and short-term (30-day) and long-term (3-year) postoperative mortality in older adults undergoing hip fracture surgery.

**Findings:**

Lower preoperative mFES scores were independently associated with increased 30-day postoperative mortality and higher long-term mortality risk during the 3-year follow-up period, although the association was stronger for short-term mortality.

**Message:**

Preoperative fear of falling may represent an important marker of psychological and functional vulnerability in older adults with hip fractures. The mFES is a brief and practical bedside tool that may help identify high-risk patients.

## Introduction

With the global population aging, the incidence of hip fractures among the older adults is increasing [1]. Geriatric hip fracture is associated with high mortality and disability rates, with 1-year mortality as high as 20–30% [2]. Despite significant advancements in surgical techniques and perioperative management, postoperative mortality in this population remains unacceptably high, posing a severe public health challenge [1]. Existing research has identified a series of objective physiological risk factors, such as advanced age, male sex, high comorbidity burden, poor nutritional status, and low preoperative functional status [3, 4], all of which point to the patient’s physiological frailty.

However, while fully addressing these physiological challenges, the potential prognostic value of a key psychological dimension—fear of falling—has not been sufficiently explored. Fear of falling is commonly assessed using validated self-report instruments, among which the Modified Falls Efficacy Scale (mFES), originally developed by Tinetti et al. and later adapted by Hill et al. [5], has been widely applied in orthopedic and geriatric populations in earlier studies. It measures perceived self-efficacy in performing daily activities without falling, rather than objective balance or physical performance. Although the Falls Efficacy Scale International (FES-I) has been recommended in more recent consensus guidelines as the preferred instrument for assessing concern about falling [6], the mFES remains a validated and frequently used measure in earlier hip fracture research [7, 8]. Studies have shown that a high level of fear of falling is associated with reduced physical activity, decreased muscle strength, social isolation, and poorer quality of life [7, 9].

However, current hip fracture research has focused predominantly on surgical complications, functional recovery, and traditional medical risk factors. Psychological dimensions are comparatively underexplored, and when assessed, attention is often limited to depression or cognitive impairment [3, 4]. Moreover, evidence regarding the prognostic significance of falls-related self-efficacy for postoperative mortality remains limited, particularly with respect to both short-term and long-term outcomes. Therefore, this study aimed to investigate the association between preoperative fear of falling, assessed using the mFES, and postoperative mortality in older adults undergoing surgery for hip fractures.

## Method

### Study design and participants

This was a post-hoc analysis utilizing data from a prospective observational study conducted in China that evaluated the impact of orthogeriatric co-management on older adults with hip fractures (ClinicalTrials.gov Identifier: NCT03184896; Registration date: June 14, 2017). The study was carried out at a tertiary care hospital in Beijing, China, which implemented a standardized orthogeriatric co-management model for patients aged ≥ 65 years with hip fractures. All patients were enrolled between November 26, 2018, and November 30, 2019.

Inclusion criteria were: (1) age 65 years or older; (2) a radiologically confirmed hip fracture (femoral neck, intertrochanteric, or subtrochanteric); (3) hospital admission within 21 days of injury; (4) receipt of surgical treatment for the fracture; and (5) completion of the mFES assessment upon admission. Exclusion criteria were: (1) a diagnosis of neoplastic pathological fracture or periprosthetic fracture; or (2) lack of informed consent.

### Assessment of fear of falling

Fear of falling was assessed using the Chinese version of mFES during the baseline evaluation at hospital admission. The mFES consists of 14 items, with each item scored from 0 (no confidence) to 10 (complete confidence). The total score is the average of all the item scores. Therefore, the total score ranges from 0 to 10, with a lower score indicating greater fear of falling.

It is worth noting that although the mFES asks patients to rate their confidence in performing daily activities that may be temporarily impossible after a hip fracture, the scale captures fall‑related self‑efficacy—a psychological construct distinct from objective physical function. Patients can still comprehend the task demands and provide a meaningful judgment based on their pre‑fracture functional status. Notably, all patients in this cohort had acute hip fractures with an injury‑to‑admission interval of ≤ 21 days, which minimizes recall bias and supports the validity of the preoperative mFES assessment in this population.

### Data collection and outcomes

Trained research nurses were responsible for collecting baseline and follow-up data. Baseline characteristics included age, sex, fracture type, American Society of Anesthesiologists (ASA) grade, comorbidities (hypertension, diabetes mellitus, coronary heart disease, stroke), pre-fracture mobility, type of surgery, length of hospital stay (LOS), time from admission to surgery.

Patients were followed up via telephone at four predefined time points after hospital admission to ascertain survival status: 30-day, 120-day, 1-year, and 3-year. The primary outcomes were 30-day postoperative mortality and long-term all-cause mortality during the 3-year follow-up period.

### Statistical analysis

Continuous variables were presented as mean ± standard deviation (SD) or median with interquartile range (IQR), as appropriate according to data distribution. Categorical variables were expressed as frequencies and percentages (n, %). To evaluate the association between preoperative fear of falling and postoperative mortality, the Modified Falls Efficacy Scale (mFES) score was analyzed as a continuous variable. Univariable and multivariable logistic regression analyses were performed to assess the association between preoperative mFES score and 30-day mortality. Variables included in the multivariable model were selected based on clinical relevance and prior literature, including age, sex, ASA grade, stroke history, and pre-fracture mobility. Results were reported as odds ratios (ORs) with corresponding 95% confidence intervals (95% CIs). To further evaluate the longitudinal association between preoperative mFES score and long-term survival, Cox proportional hazards regression analyses were performed. Univariable and multivariable Cox models were constructed using the same covariates as those included in the logistic regression analysis. Hazard ratios (HRs) with 95% CIs were reported. The proportional hazards assumption was assessed using Schoenfeld residuals and verified using the global test based on the correlation between scaled Schoenfeld residuals and follow-up time. No significant violation of the proportional hazards assumption was detected. All analyses were performed using IBM SPSS Statistics version 25.0 (IBM Corp., Armonk, NY, USA) and R software (version 4.5.2; R Foundation for Statistical Computing, Vienna, Austria). A two-sided P-value < 0.05 was considered statistically significant.

## Result

### Study population

**Fig. 1:**
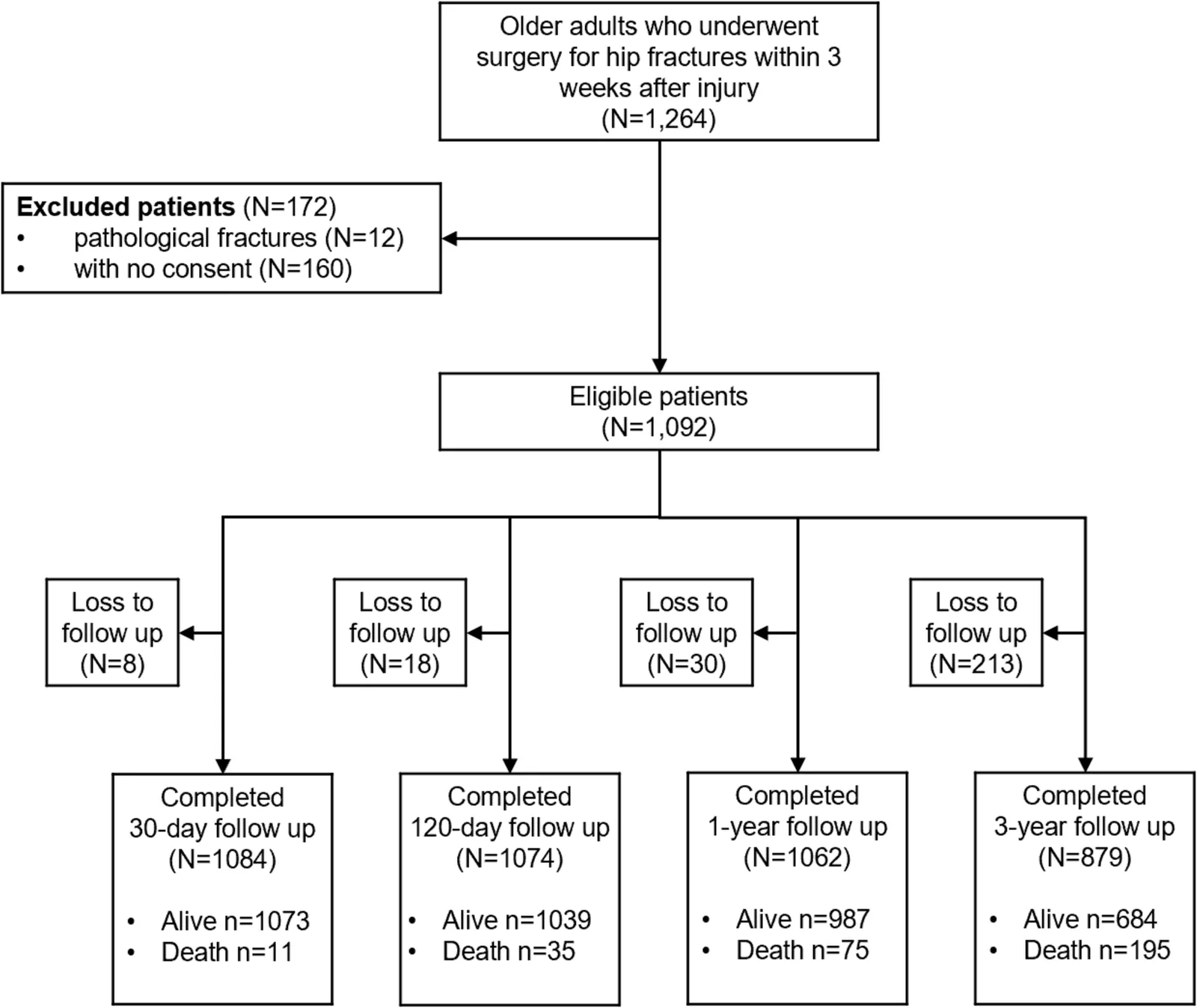
Flowchart of patient inclusion and follow-up

During the study period, a total of 1092 older adults aged 65 years or older who underwent surgery for hip fractures were enrolled in the study. Follow-up data were available for 1084 patients at 30-day follow up, 1074 patients at 120-day follow up, 1062 patients at 1-year follow up, and 879 patients at 3-year follow up. The participant inclusion process and follow-up status are presented in Fig. 1.

#### Baseline characteristics

The baseline demographic and clinical characteristics of the study cohort are presented in Table 1. The mean age of the cohort was 80.18 ± 7.79 years, and 72.0% of participants were female. The median preoperative mFES score was 8.04 (IQR 5.75–10.00). Hypertension was present in 57.1% of patients, diabetes mellitus in 27.7%, coronary heart disease in 22.3%, and stroke in 17.8%. Regarding overall physical status, 38.2% of patients were classified as ASA grade 3–4.

**Table 1:** Baseline demographic and clinical characteristics of the study cohort

	Total (n = 1092)
mFES score/point, median (IQR)	8.04 (5.75, 10.00)
Age/years, mean ± SD	80.18 ± 7.79
Sex, n (%)	
Female	786 (72.0)
Male	306 (28.0)
Hypertension, n (%)	623 (57.1)
Diabetes mellitus, n (%)	303 (27.7)
Coronary heart disease, n (%)	244 (22.3)
Stroke, n (%)	194 (17.8)
ASA grade, n (%)	
Grade 1–2	675 (61.8)
Grade 3–4	417 (38.2)
Pre-fracture mobility, n (%)	
Independent	765 (70.1)
Walking aid	307 (28.1)
Non-ambulant	20 (1.8)
Fracture type, n (%)	
Femoral neck fracture	556 (50.9)
Intertrochanteric fracture	511 (46.8)
Subtrochanteric fracture	25 (2.3)
Type of surgery, n (%)	
Dynamic hip screw	3 (0.3)
Cannulated screws	111 (10.2)
Cephalomedullary nail	531 (48.6)
Hemiarthroplasty	326 (29.9)
Total hip arthroplasty	121 (11.1)
Time from admission to surgery/day, median (IQR)	2.18 (1.51, 3.08)
Length of hospital stay/day, median (IQR)	5.04 (4.00,6.58)

*mFES* the Modified Falls Efficacy Scale, *ASA* American Society of Anesthesiologists score

Most patients (70.1%) were independently ambulatory before fracture, whereas 28.1% required walking aids and 1.8% were non-ambulatory. Femoral neck fractures accounted for 50.9% of cases, followed by intertrochanteric fractures (46.8%) and subtrochanteric fractures (2.3%). Cephalomedullary nailing was the most commonly performed surgical procedure (48.6%), followed by hemiarthroplasty (29.9%). The median time from admission to surgery was 2.18 days (IQR 1.51–3.08), and the median length of hospital stay was 5.04 days (IQR 4.00–6.58).

#### Association between preoperative mFES score and short‑term and mid‑term mortality

The associations between preoperative mFES score and short-term as well as mid-term postoperative mortality are summarized in Table 2. In the univariable logistic regression analyses, higher mFES scores were significantly associated with lower odds of mortality at 30 days (OR 0.760, 95% CI 0.614–0.941, P = 0.012), 120 days (OR 0.837, 95% CI 0.739–0.948, P = 0.005), and 1 year (OR 0.863, 95% CI 0.790–0.943, P = 0.001). After adjustment, the association remained statistically significant only at 30 days: each 1-point increase in mFES score was associated with a 22.0% reduction in the odds of 30-day mortality (adjusted OR 0.780, 95% CI 0.625–0.974, P = 0.028). At 120 days and 1 year, the associations did not reach statistical significance (adjusted OR 0.878, 95% CI 0.765–1.008, P = 0.065; and adjusted OR 0.917, 95% CI 0.831–1.012, P = 0.086, respectively), although a consistent trend toward lower mortality with higher mFES scores was observed across all time points, with the strongest effect at 30 days and gradual attenuation thereafter.

**Table 2:** Association between preoperative mFES score and short‑term and mid‑term mortality: logistic regression analysis

	Event	Unadjusted OR (95% CI)	*P* value	Adjusted OR (95% CI)	Adjusted *P* value*
30-day mortality, n (%)	11 (1.0)	0.760 (0.614–0.941)	0.012	0.780 (0.625–0.974)	0.028
120-day mortality, n (%)	35 (3.3)	0.837 (0.739–0.948)	0.005	0.878 (0.765–1.008)	0.065
1-year mortality, n (%)	75 (7.1)	0.863 (0.790–0.943)	0.001	0.917 (0.831, 1.012)	0.086

*Adjusted for age, sex, ASA grade, stroke history, and pre-fracture mobility

#### Association between preoperative mFES score and long-term mortality

The associations between preoperative mFES score and long-term mortality are presented in Table 3. In the univariable Cox proportional hazards analysis, higher mFES scores were significantly associated with a lower hazard of death during the 3-year follow-up period (HR 0.868, 95% CI 0.824–0.915, *P* < 0.001). After adjustment for age, sex, ASA grade, stroke history, and pre-fracture mobility, higher mFES scores remained independently associated with lower long-term mortality risk (adjusted HR 0.925, 95% CI 0.874–0.979, *P* = 0.007). The proportional hazards assumption was assessed using Schoenfeld residuals, and no significant violation was detected (global test: P = 0.69). Each 1-point increase in mFES score corresponded to an approximately 7.5% reduction in the long-term hazard of death following hip fracture surgery.

**Table 3:** Association between preoperative mFES score and long-term mortality: Cox proportional hazards analysis

	Events	Unadjusted HR (95% CI)	*P* value	Adjusted HR (95% CI)	Adjusted *P* value*
3-year mortality, n (%)	195 (17.8)	0.868 (0.824–0.915)	< 0.001	0.925 (0.874–0.979)	0.007

*Adjusted for age, sex, ASA grade, stroke history, and pre-fracture mobility

## Discussion

This study demonstrated that lower preoperative mFES scores were independently associated with both increased short-term and long-term postoperative mortality in older adults with hip fractures. Specifically, higher fear of falling was associated with a substantially higher risk of 30-day mortality, while the association with long-term mortality over the 3-year follow-up period remained statistically significant but attenuated after multivariable adjustment. These findings suggest that preoperative fear of falling may represent not only an acute psychological stress response, but also a broader marker of vulnerability influencing postoperative survival trajectories.

Growing evidence indicates that psychological factors contribute importantly to outcomes after hip fracture surgery. Previous studies have shown that depression, cognitive impairment, and psychological frailty are associated with increased mortality, poorer functional recovery, and reduced quality of life in older adults with hip fractures [10–12]. The present study extends this literature by focusing specifically on fear of falling, a highly prevalent but often underrecognized psychological concern in geriatric orthopedic patients. Prior studies have demonstrated that fear of falling is closely associated with reduced mobility, physical inactivity, sarcopenia, and social isolation [13, 14].

The mechanisms underlying the associations between preoperative mFES score and postoperative mortality may differ between the short-term and long-term postoperative periods. The stronger association observed with 30-day mortality may reflect the acute impact of psychological vulnerability during the perioperative phase. Higher fear of falling may contribute to heightened stress responses, reduced participation in early mobilization, poorer adherence to rehabilitation, and increased fear-driven activity restriction immediately after surgery. These factors may increase the risk of early postoperative complications such as pneumonia, venous thromboembolism, delirium, and cardiopulmonary events, thereby contributing to increased short-term mortality. In addition, lower mFES scores are closely associated with frailty, impaired mobility, and poorer baseline functional reserve [15], all of which are established predictors of adverse early postoperative outcomes after hip fracture surgery.

In contrast, although the association between mFES score and mortality remained significant in the long-term Cox model, the effect size was attenuated. This finding suggests that preoperative fear of falling may also reflect broader and more persistent vulnerability beyond the immediate postoperative period. Patients with higher fear of falling often experience sustained reductions in physical activity, progressive functional decline, social isolation, and reduced engagement in rehabilitation and secondary prevention strategies. Over time, these factors may contribute cumulatively to poorer overall health status and increased long-term mortality risk. However, as follow-up duration increases, long-term survival is increasingly influenced by multiple competing factors, including chronic comorbidities, aging-related physiological decline, malignancy, and socioeconomic determinants. Consequently, the relative contribution of a single preoperative psychological factor becomes less pronounced over time, which may explain the weaker association observed in the adjusted Cox analysis.

From a clinical perspective, these findings support the incorporation of preoperative fear-of-falling assessment into routine orthogeriatric evaluation. The mFES is a brief and easy-to-administer instrument that may help identify psychologically vulnerable patients at increased risk of postoperative mortality. Given that fear of falling is potentially modifiable, early multidisciplinary interventions involving geriatricians, rehabilitation specialists, nursing staff, and psychological support services may be beneficial for improving perioperative management and postoperative recovery in this high-risk population [16–18].

Several limitations should be acknowledged. First, this study was a post-hoc analysis of a prospective cohort, and the assessment of fear of falling was limited to the mFES. Although the FES-I is currently recommended in international guidelines as the preferred instrument for assessing concern about falling, during the period of data collection (2018–2019) it had not yet achieved widespread implementation or strong recommendation in routine clinical practice in our setting. In addition, the mFES primarily evaluates self-perceived efficacy in performing daily activities rather than concern or fear about falling itself, and its interpretation may be influenced by the acute functional limitations associated with hip fracture. However, all participants were assessed within 21 days after injury, which likely reduced recall bias regarding pre-fracture functional confidence. Furthermore, the mFES was assessed only once at admission, precluding evaluation of postoperative changes and longitudinal trajectories of fear of falling. Despite these limitations, the mFES has been widely used in previous hip fracture and geriatric studies and remains a practical tool for identifying psychological vulnerability in older adults with hip fractures. Second, we did not systematically assess other potentially relevant psychological or cognitive variables, such as depression, anxiety, personality traits, or detailed cognitive impairment measures. These factors may correlate with fear of falling and could partially mediate or confound the observed association between low mFES scores and postoperative mortality. Future studies incorporating multidimensional psychological and cognitive assessments are warranted to better elucidate these complex interrelationships. Third, as a single-center observational study, the generalizability of our findings to other settings should be interpreted with caution. In addition, unmeasured clinical factors—such as rehabilitation intensity, time to weight-bearing, and variations in surgical technique—may also have influenced postoperative outcomes despite multivariable adjustment. Fourth, despite systematic follow-up, the attrition rate at the 3-year follow-up (approximately 19.5%) may affect the stability of the long-term mortality estimates. Finally, the study primarily focused on all-cause mortality. Future research could incorporate repeated assessments using validated instruments such as the FES-I to further explore cause-specific mortality, re-fracture rates, and functional recovery outcomes, thereby providing a more comprehensive evaluation of the prognostic relevance of fear of falling.

## Conclusion

Lower preoperative mFES scores were independently associated with increased postoperative mortality in older adults undergoing hip fracture surgery. The association was strongest for 30-day mortality, while a weaker but persistent association with long-term mortality was also observed over the 3-year follow-up period.

## Data Availability

The datasets used in this study are not publicly available because of patient confidentiality but are available from the corresponding author on reasonable request.

## Abbreviations

mFES: The Modified Fall Effectiveness Scale
ASA: The American Society of Anesthesiologists
OR: Odds ratio
95% CI: 95% Confidence interval
SD: Standard deviation
IQR: Interquartile range
HR: Hazard ratio
